# Decreased Mdm2 Expression Inhibits Tumor Development and Extends Survival Independent of Arf and Dependent on p53

**DOI:** 10.1371/journal.pone.0046148

**Published:** 2012-09-28

**Authors:** Christine M. Eischen, Kelli Boyd

**Affiliations:** Department of Pathology, Microbiology and Immunology, Vanderbilt University Medical Center, Nashville, Tennessee, United States of America; Rush University Medical Center, United States of America

## Abstract

Inactivation of the Arf-Mdm2-p53 tumor suppressor pathway is a necessary event for tumorigenesis. Arf controls Mdm2, which in turn regulates p53, but Arf and Mdm2 also have p53-independent functions that affect tumor development. Moreover, inhibition of oncogene-induced tumorigenesis relies on Arf and p53, but the requirements of Arf and p53 in tumor development initiated in the absence of overt oncogene overexpression and the role of Mdm2 in this process remain unclear. In a series of genetic experiments in mice with defined deficiencies in Arf, Mdm2 and/or p53, we show Mdm2 haploinsufficiency significantly delayed tumorigenesis in mice deficient in Arf and p53. *Mdm2* heterozygosity significantly inhibited tumor development in the absence of Arf, and in contrast to Myc oncogene-driven cancer, this delay in tumorigenesis could not be rescued with the presence of one allele of *Arf*. Notably, Mdm2 haploinsufficieny blocked the accelerated tumor development in Arf deficient mice caused by *p53* heterozygosity. However, tumorigenesis was not inhibited in *Mdm2* heterozygous mice lacking both alleles of *p53* regardless of *Arf* status. Surprisingly, loss of Arf accelerated tumor development in *p53*-null mice. Tumor spectrum was largely dictated by *Arf* and *p53* status with Mdm2 haploinsufficiency only modestly altering the tumor type in some of the genotypes and not the number of primary tumors that arose. Therefore, the significant effects of Mdm2 haploinsufficiency on tumor latency were independent of Arf and required at least one allele of *p53*, and an Mdm2 deficiency had minor effects on the types of tumors that developed. These data also demonstrate that decreased levels of Mdm2 are protective in the presence of multiple genetic events in *Arf* and *p53* genes that normally accelerate tumorigenesis.

## Introduction

The Mdm2 oncogene is an essential regulator of tumorigenesis in part due to its control over the tumor suppressor p53, which is inactivated in half of all human malignancies [1], [2]. Mdm2 binds to p53 and inhibits its transcriptional activity. As an E3 ubiquitin ligase, Mdm2 also ubiquitinates p53 and thereby targets p53 for destruction. Arf, the second protein encoded in the Ink4a locus, is a regulator of Mdm2 [3]. Arf binds to Mdm2 and blocks Mdm2 from inhibiting p53. Arf is frequently inactivated in human malignancies. The Arf-Mdm2-p53 tumor suppressor pathway itself is activated by cellular stress, such as hyperproliferative signals from oncogenes, DNA damage, hypoxia, and many more, resulting in apoptosis, cell cycle block, or senescence, which protects genome integrity and inhibits tumorigenesis [4]–[6].

Deletion of *p53* or *Arf* in mice results in tumor development with 100% penetrance [7], [8], cementing their roles as tumor suppressors. *p53*-null mice have an average life span of six months [8], whereas the mean life span of an *Arf−/−* mice is ten months [7]. Thymic T cell lymphomas or sarcomas develop in *p53−/−* mice [8], whereas *Arf*-null mice have a slightly different tumor spectrum. Mice lacking Arf usually develop a splenic lymphoma (B or T cell) or a sarcoma [7]. Both *p53−/−* and *Arf−/−* mice rarely develop carcinoma. Sarcomas are reported to be the primary tumor type that develops in C57Bl/6X129Sv mixed *Arf*-null mice [9], but other studies have shown lymphomas predominate in *Arf−/−* mice with a C57Bl/6 background [10], [11]. Mice lacking both Arf and p53 are reported to have a mean survival equal to that of *p53−/−* mice [12]. However, 28% of the *Arf−/−p53−/−* mice developed more than one primary tumor type, typically a lymphoma and a sarcoma, whereas a single tumor type typically emerges in both *Arf−/−* and *p53−/−* mice. In addition, *Arf−/−p53−/−* mice that also lack both alleles of *Mdm2*, tumor latency is unaltered, but 47% of the mice developed more than one primary tumor type [12]. Therefore, complete loss of Mdm2 has no effect on the survival of *Arf/p53*-null mice, but does alter the tumor spectrum.

It was previously reported that deletion of both alleles of *Arf* does not cooperate to accelerate tumorigenesis in mice with a bialleleic deletion of *p53* [12], but deletion of *Arf* did cooperate with overexpression of Mdm2 to accelerate tumorigenesis [11]. These latter data indicate that the effects of Mdm2 on tumor development may not be entirely dependent on p53. Notably, overexpression of Mdm2 changes tumor spectrum but not tumor latency in *p53−/−* mice [13]. Another study showed *Mdm2+/−p53−/−* mice had a six day longer average survival and had a higher incidence of sarcomas compared to *p53*-null only and *Mdm2/p53*-double null mice [14]. These results suggested *Mdm2* haploinsufficiency could affect tumor development and type in the absence of p53. We have observed a significant delay in tumor development in *Mdm2+/−Arf−/−* mice as compared to *Mdm2+/+Arf−/−* mice, but tumor spectrum was similar between these two genotypes [10]. Although our study indicates *Mdm2* heterozygosity can influence tumor development in the presence of p53, we did not test whether this outcome on tumorigenesis of Mdm2 haploinsufficiency was dependent on p53. In this manuscript, we examine the effects of decreased levels of Mdm2 on tumor development and the contribution of Arf and p53. We show the delay in tumor development caused by an Mdm2 haploinsufficiency occurs in Arf deficient mice and mice with one allele of *p53*, but not in mice lacking both alleles of *p53*. Notably, the data demonstrate Arf does not regulate Mdm2 during tumor development in the absence of oncogene overexpression. Unexpectedly, loss of *Arf* cooperated with deletion of *p53* to accelerate tumorigenesis. The tumor spectrum was only modestly altered in some of the genotypes of mice when *Mdm2* was heterozygous. Therefore, the negative effects of Mdm2 haploinsufficiency on tumorigenesis are significant, require at least one allele of *p53*, and can occur in the background of an Arf deficiency.

## Results

### Increased Tumor Latency in Mdm2+/−Arf−/− Mice is Dependent on p53

**Figure 1:**
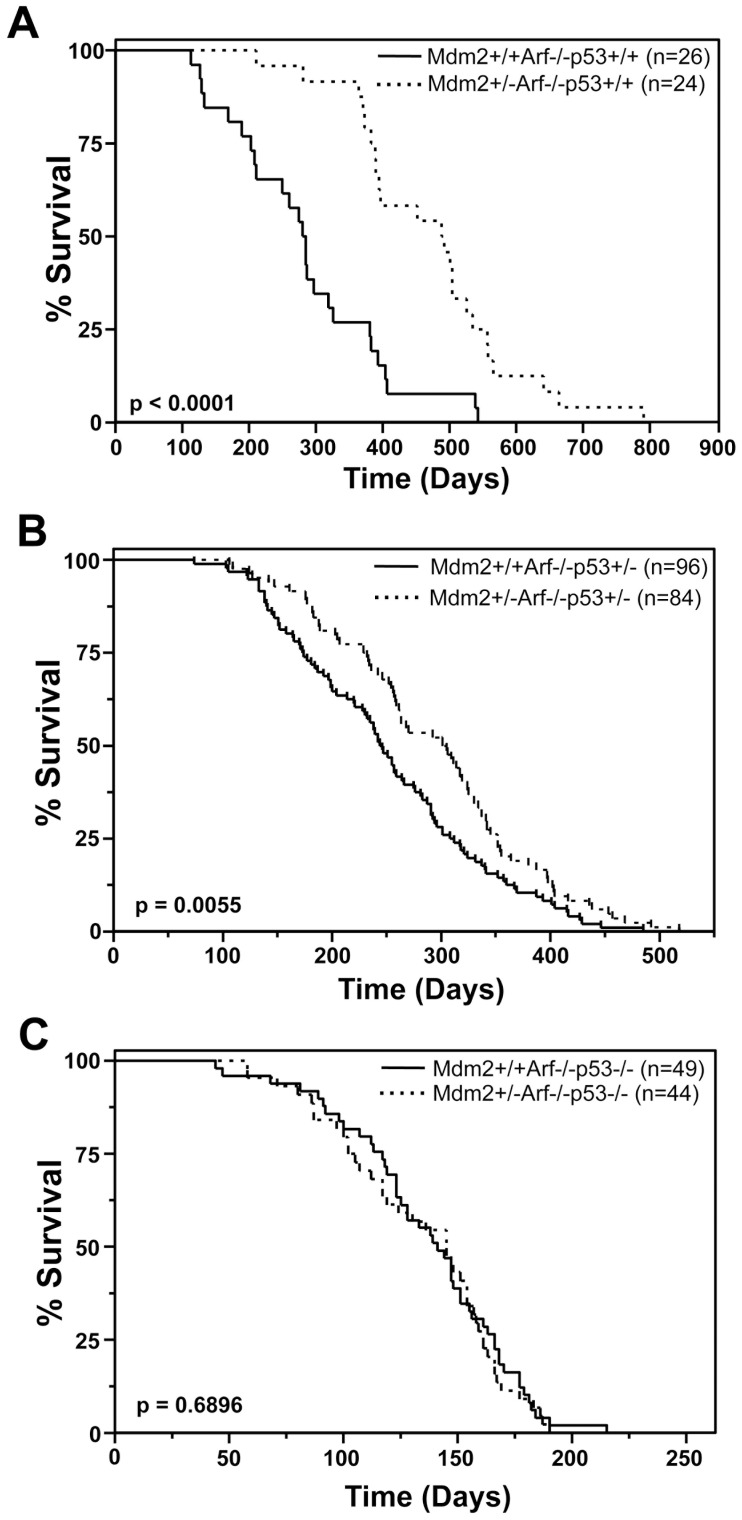
The contribution of p53 to the delay in tumorigenesis of *Arf−/−* mice caused by *Mdm2* heterozygosity. Kaplan-Meier survival curves of the indicated genotype of mice. The numbers of mice in each group are denoted by the *n* values. P values calculated by log-rank test.

We previously reported in mice with biallelic deletion of *Arf*, *Mdm2* heterozygosity profoundly inhibited tumorigenesis and significantly extended survival over that of mice that were only *Arf*-null [10]. We repeated this on a new cohort of mice and obtained similar results (Fig. 1A, Table 1); however, it is unclear what role p53 has in this delay in tumor development attributed to an Mdm2 haploinsufficiency. Moreover, alterations in the levels of Arf, Mdm2, or p53 can significantly change tumor development, but the impact of altered levels of these genes on each other during tumor development is less understood. By using a genetic approach, we addressed these issues in this study. Firstly, to determine the requirement of p53 in mediating the delay in tumorigenesis in *Mdm2+/−Arf*−/− mice and the effects of altering the gene dosage of p53, we generated *Mdm2+/−Arf*−/− mice and littermate *Mdm2+/+Arf*−/− controls lacking one allele of *p53* and monitored them for tumor development. *Mdm2* heterozygous *Arf−/−p53+/−* mice had a significantly protracted rate of tumor development compared to *Mdm2* wild-type *Arf−/−p53+/−* mice, resulting in increased survival (Fig. 1B). The mean survival of *Mdm2+/−Arf−/−p53+/−* mice (292 days) was significantly longer than the mean survival of *Mdm2+/+Arf−/−p53+/−* mice (251 days; p = 0.0055 log-rank test, Table 1). Therefore, an Mdm2 haploinsufficiency in *Arf*-null mice still delays tumor development with loss of one allele of *p53*, indicating that both alleles of *p53* are not required for this delay in tumorigenesis. These data also show that loss of one allele of *p53* is insufficient to rescue the effects of *Mdm2* haploinsufficiency.

**Table 1:** Mean and median survivals of mice.

Genotype	*Mdm2+/+*	*Mdm2+/−*	p value*
*Arf+/+p53−/−*	164, 154 (39)	167, 174 (42)	0.3923
*Arf+/−p53−/−*	154, 151 (25)	149, 146 (40)	0.3477
*Arf−/−p53−/−*	137, 141 (49)	134, 145 (44)	0.6896
*Arf−/−p53+/−*	251, 245 (96)	292, 303 (84)	0.0055
*Arf−/−p53+/+*	285, 283 (26)	472, 490 (24)	<0.0001
*Arf+/−p53+/−*	376, 407 (32)	450, 467 (29)	0.0115

Values are listed as mean, median and number of mice in parentheses.

* log-rank tests of *Mdm2+/+* compared to *Mdm2+/−* mice.

If p53 is mediating the delay in tumorigenesis from an Mdm2 haploinsufficiency, one allele of *p53* may be sufficient for these effects in *Mdm2+/−Arf−/−p53+/−* mice. To test whether Mdm2 haploinsufficiency would still inhibit tumor development in the absence of p53, we evaluated tumorigenesis in *Arf−/−* mice that also lacked both alleles of *p53*. *Mdm2+/−Arf−/−p53−/−* mice and littermate *Mdm2+/+Arf−/−p53−/−* controls had analogous rates of tumor development resulting in similar Kaplan-Meier survival curves (Fig. 1C). The *Mdm2+/−Arf*−/−*p53−/−* mice had a 134 day mean survival, and the *Mdm2+/+Arf−/−p53−/−* mice had a mean survival of 137 days (Table 1). The difference in their survivals was not statistically significant (p = 0.6896, log-rank test), indicating that complete loss of p53 abrogated the effects of Mdm2 haploinsufficiency in tumor development in *Arf*-null mice. Therefore, the presence of at least one allele of *p53* is required to mediate the effects of Mdm2 haploinsufficiency on tumor development in the absence of Arf.

### Tumor Latency is Dictated by p53 Gene Dosage

**Figure 2:**
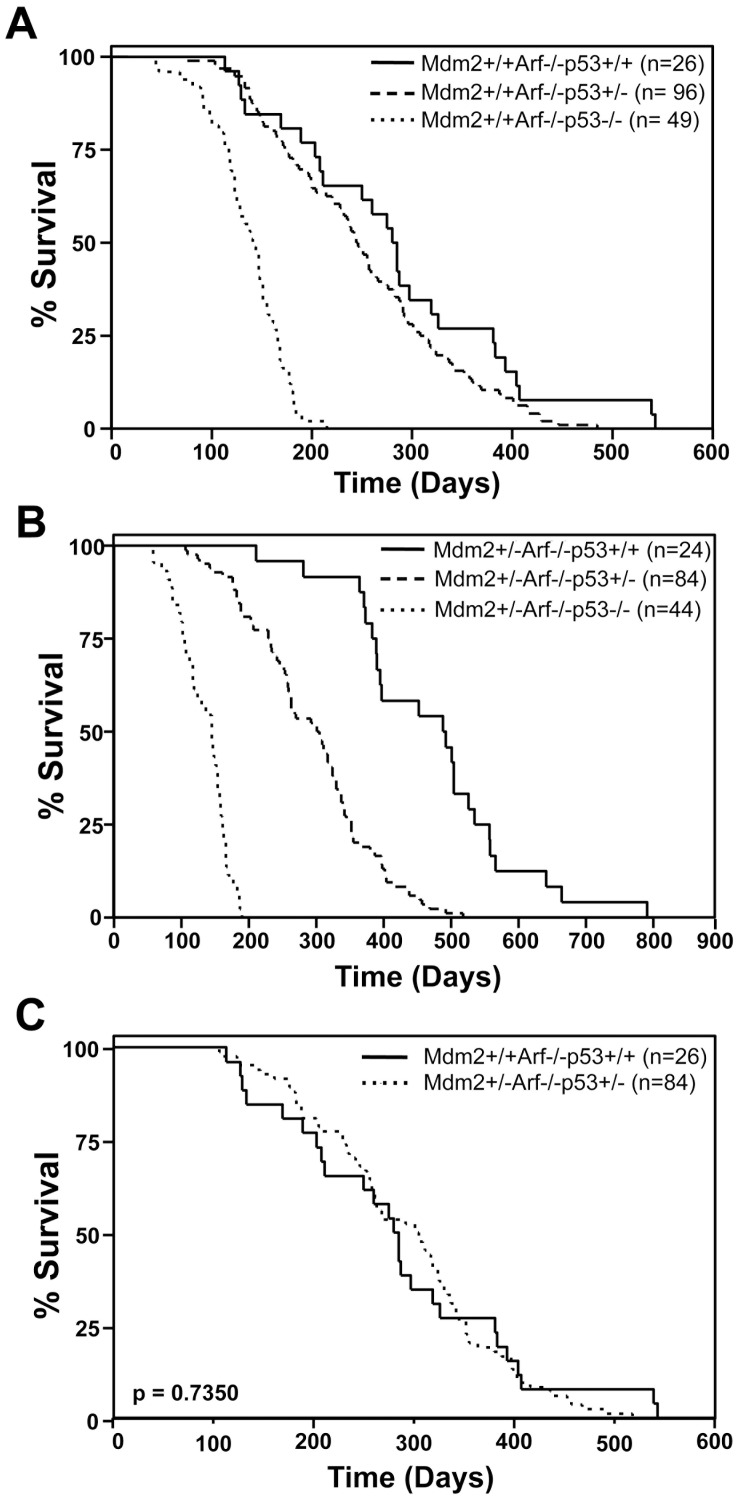
The gene dosage effect of *p53* on the survival of *Mdm2* wild-type and *Mdm2* heterozygous mice in the absence of Arf. Kaplan-Meier survival curves of the indicated genotype of mice. The numbers of mice in each group are denoted by the *n* values. P value calculated by log-rank test.

It is well established that loss of functional p53 accelerates tumor development [6]. This occurs even in an *Arf*-null background [11], [12], indicating loss of p53 is dominant. As previously reported [11], [12], *Arf*-null mice lacking two alleles of *p53* have a shorter survival than *Arf*-null mice that had one or both alleles of *p53* (Fig. 2A). We observed *Arf*−/−*p53+/−* mice had an increased rate of tumor development compared to *Arf*−/−*p53+/+* mice (251 versus 285 days mean survival; Table 1). Since *Mdm2* heterozygosity greatly delays tumor development in *Arf*-null mice that have both alleles of *p53*, the difference in mean survival between *p53+/+* and the *p53+/− Arf*-null *Mdm2+/−*mice was much larger than their *Mdm2* wild-type counterparts and quite significant (p<0.0001, log-rank test; Fig. 2B, Table 1). Notably, mice that were *Mdm2+/−Arf*−/−*p53+/−* had a very similar mean survival as *Arf−/−* mice (292 versus 285 days, p = 0.7350 log rank test; Fig. 2C, Table 1). The data indicate that in the absence of Arf, Mdm2 haploinsufficiency compensated for a single genetic hit to *p53* resulting in a rate of tumor development analogous to mice with two wild-type *p53* alleles. These results highlight the important role Mdm2 has in tumorigenesis and the protective role lower levels of Mdm2 can have on cells predisposed to developing cancer.

### Arf does not Regulate Mdm2 during Tumorigenesis in the Absence of Oncogene Pressure

**Figure 3:**
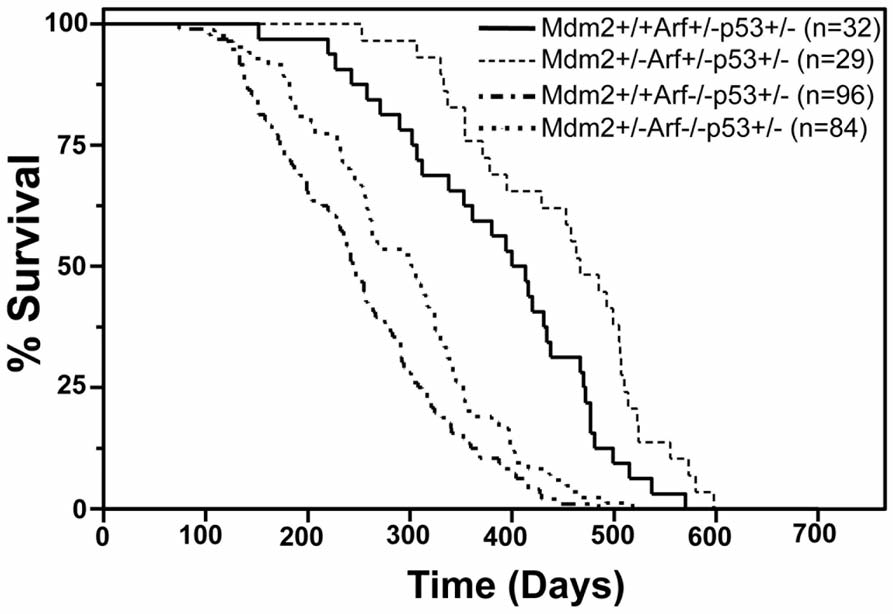
Arf heterozygosity does not rescue the Mdm2 haploinsufficiency induced delay in tumor development. Kaplan-Meier survival curves of the indicated genotype of mice. The numbers of mice in each group are denoted by the *n* values.

Previously, we reported in Myc oncogene-driven cancer, loss of one allele of *Arf* rescued the delay in tumorigenesis caused by *Mdm2* heterozygosity, demonstrating Arf levels regulate Mdm2 during oncogene-induced tumorigenesis [15]. Data above show that in the absence of Arf, *Mdm2* heterozygosity inhibited tumor development when at least one allele of *p53* was present (Fig. 1B). To assess the influence Arf expression has on Mdm2 in the absence of overt oncogene overexpression, we generated, and followed for tumor development, *p53* heterozygous mice that were *Mdm2* wild-type or heterozygous and with only one allele of *Arf*. *Mdm2+/−Arf+/−p53+/−* mice had a significantly longer mean survival than *Mdm2+/+Arf+/−p53+/−* mice (450 versus 376 days, p = 0.0115 log-rank test, Table 1 and Fig. 3). Therefore, *Arf* heterozygosity in *Mdm2+/−p53+/−* mice did not restore the rate of tumorigenesis to that of *Mdm2+/+Arf+/−p53+/−* mice. In addition, although Mdm2 haploinsufficiency inhibits tumor development in *Arf−/−p53+/−* mice, these mice still have a mean survival that is significantly shorter than *Mdm2+/+Arf+/−p53+/−* (Fig. 3, Table 1), indicating biallelic loss of Arf is dominant in this situation. Therefore, although Arf is a known regulator of Mdm2 [3], Arf levels do not appear to regulate Mdm2 in non-oncogene driven cancers.

### Arf Alters Tumor Latency in Mice Lacking p53 Irrespective of Mdm2 Heterozygosity

**Figure 4:**
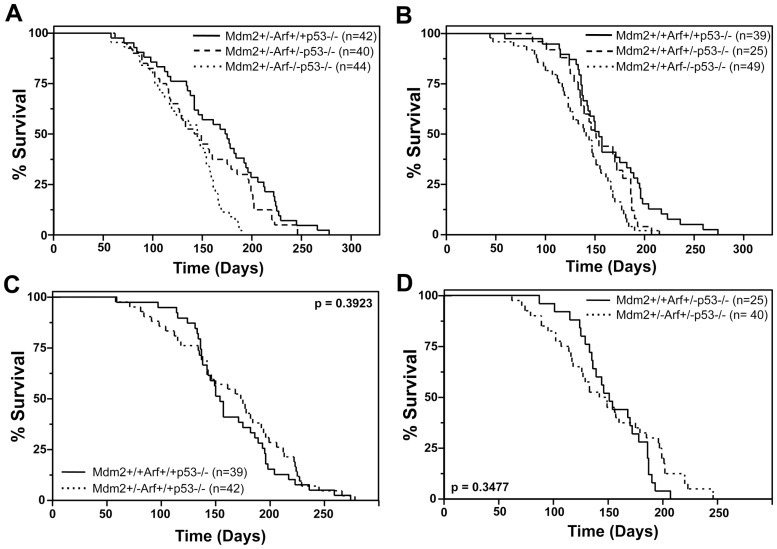
The gene dosage of *Arf* contributes to tumorigenesis in the absence of *p53*. Kaplan-Meier survival curves of the indicated genotype of mice. The numbers of mice in each group are denoted by the *n* values. P values calculated by log-rank test.

As a tumor suppressor, Arf deficiency leads to tumorigenesis in a gene dosage dependent fashion. Specifically, *Arf* heterozygous mice have an extended survival over that of *Arf*-null mice, but a reduced survival compared to *Arf* wild-type mice [9]. Deletion of both alleles of *Arf* also significantly accelerates tumor development in *p53* heterozygous mice (Fig. 3 and [11]). However, loss of only one allele of *Arf* does not alter the rate of tumorigenesis in *p53+/−* mice [11], indicating that *p53* heterozygosity is dominant in this situation. Arf also has p53 independent functions that can influence tumorigenesis [16], but its contribution to tumor latency is reported to be dependent on p53 [12]. To determine whether Arf expression influences tumor development in the context of Mdm2 haploinsufficiency and independent of p53, we generated and evaluated *Mdm2+/−p53−/−* mice that had one, both, or no alleles of *Arf* and *Mdm2+/+* littermate controls. Unexpectedly, we observed that *Mdm2* heterozygous mice that were *Arf+/+p53−/−* mice had a longer mean survival than *Arf+/−p53−/−* and *Arf−/−p53−/− Mdm2* heterozygous mice (Fig. 4A, Table 1). The difference in survival between *Arf+/+* and *Arf+/− Mdm2+/−p53−/−* mice was not statistically significant (p = 0.1413, log-rank test), but the difference in survival between *Arf+/+* and *Arf−/− Mdm2+/−p53−/−* mice was statistically significant (p<0.0001, log-rank test). Since a previous study did not report a difference in survival between *Arf+/+p53−/−* and *Arf−/−p53−/−* mice [12], we questioned whether the accelerated tumor development in *p53−/−* mice with loss of *Arf* was somehow due to the *Mdm2* heterozygosity. Surprisingly however, a similar trend was observed for the *Mdm2* wild-type *p53*-null mice with loss of *Arf* accelerating tumorigenesis (Fig. 4B, Table 1). The difference in survival between *Mdm2+/+Arf+/+p53−/−* and *Mdm2+/+Arf−/−p53−/−* mice was significant (p = 0.0011, log-rank test), suggesting loss of *Arf* cooperated with deletion of *p53* to decrease tumor latency. We also compared the survival of mice that were *Mdm2* wild-type to *Mdm2+/−* in this cross. *Mdm2+/+Arf+/+p53−/−* mice had a similar mean survival as *Mdm2+/−Arf+/+p53−/−* (164 days versus 167 days) and this difference was not statistically significant (p = 0.3923, log-rank test, Table 1, Fig. 4C). In addition, the mean survivals were similar between *Mdm2+/−Arf+/−p53−/−* (149 days) and *Mdm2+/+Arf+/−p53−/−* (154 days) mice (p = 0.3477, log-rank test, Table 1, Fig. 4D). The data are consistent with our findings above in that tumor latency was not altered with Mdm2 haploinsufficiency in *p53*-null mice regardless of *Arf* status. However, our results unexpectedly show an Arf deficiency accelerates tumor development in *p53*-null mice, indicating that Arf loss has p53-independent functions that contribute to tumor latency.

### Tumor Spectrum is Altered by Mdm2 Haploinsufficiency in Some Genotypes

In addition to tumor latency, tumor spectrum is also a measure of whether a gene or dosage of a gene affects tumorigenesis. Alterations in tumor spectrum can reveal functions of a gene even when the rate of tumor development is unaltered. Therefore, we performed a histopathological evaluation of the tumors that developed in each of the genotypes of mice in this study; a total of 195 mice were evaluated. In both *Mdm2+/+* and *Mdm2+/−* mice of different *Arf* and/or *p53* deficiencies analyzed, the expected tumor types arose. Neither gender nor age determined the type of malignancy that developed for any of the genotypes. Lymphomas and sarcomas predominated with rare carcinomas arising (Table 2). The lymphomas that arose had numerous mitotic figures and the classic organ involvement (e.g., lymph nodes and spleen) with tumor infiltrates into organs, such as the liver and lungs. There were five subtypes of sarcomas that developed in the mice: hemangio, histiocytic, osteo, soft tissue, and carcino (Table 3). Soft tissue sarcomas included fibro, leiomyo, myxo, pleiomorphic, and spindle cell subtypes. The carcinomas that arose were most commonly adenocarcinomas of the lung. Very rarely other tumor types of the brain/central nervous system, such as medulloblastoma, ependymoma, and meningioma, were detected. In a small subset of mice, benign tumors (adenoma or leiomyoma) were observed.

**Table 2:** Comparison of tumor spectrum in *Mdm2+/+* and *Mdm2+/−* mice with deficiencies in *Arf* and/or *p53.*

	m+/+	m+/−	m+/+	m+/−	m+/+	m+/−	m+/+	m+/−	m+/+	m+/−
Tumor type(s)	a+/+p−/−	a+/+p−/−	a+/−p−/−	a+/−p−/−	a−/−p−/−	a−/−p−/−	a−/−p+/−	a−/−p+/−	a+/−p+/−	a+/−p+/−
*One tumor type:*	17 (89%)	16 (80%)	12 (71%)	17 (81%)	15 (75%)	14 (74%)	19 (76%)	18 (78%)	13 (81%)	12 (80%)
Lymphoma	8 (42%)	7 (35%)	5 (29%)	12 (57%)	4 (20%)	10 (53%)	3 (12%)	4 (17%)	1 (6%)	2 (13%)
Sarcoma	8 (42%)	8 (40%)	5 (29%)	5 (24%)	10 (50%)	4 (21%)	13 (52%)	11 (48%)	12 (75%)	6 (40%)
Carcinoma	1 (5%)	1 (5%)	2 (12%)	–	–	–	2 (8%)	2 (9%)	–	3 (20%)
Brain/Central Nervous System	–	–	–	–	1 (5%)	–	1 (4%)	1 (4%)	–	–
Adenoma	–	–	–	–	–	–	–	–	–	1 (7%)
*More than one tumor type:*	2 (11%)	4 (20%)	5 (29%)	4 (19%)	5 (25%)	5 (26%)	6 (24%)	5 (22%)	3 (19%)	3 (20%)
Lymphoma										
+ Sarcoma	2 (11%)	2 (13%)	5 (29%)	4 (19%)	3 (15%)	3 (16%)	2 (8%)	2 (8%)	–	–
+2 distinct Sarcomas	–	–	–	–	1 (5%)	–	–	–	–	–
+ Carcinoma	–	1 (6%)	–	–	–	–	1 (4%)	–	–	–
+ Hemangioma	–	–	–	–	–	1 (5%)	–	–	–	–
Sarcoma										
+ Carcinoma	–	–	–	–	–	–	2 (8%)	1 (4%)	1 (6%)	–
+2^nd^ distinct Sarcoma	–	–	–	–	1 (5%)	1 (5%)	–	1 (4%)	–	2 (13%)
+ Adenoma	–	1 (6%)	–	–	–	–	–	1 (4%)	1 (6%)	1 (7%)
+ Leiomyoma	–	–	–	–	–	–	1 (4%)	–		–
2 Sarcomas + Adenoma	–	–	–	–	–	–	–	–	1 (6%)	–
Number of mice analyzed:	19(7F,12M)	20(6F,14M)	17(7F,10M)	21(8F,13M)	20(10F,10M)	19(10F,9M)	25(12F,13M)	23(11F,12M)	16(8F,8M)	15(5F,10M)

Percentages were calculated from the total number of mice analyzed; – indicates no tumors of that type; F = female, M = male.

**Table 3:** Sarcoma subtype primarily dictated by Arf and p53 and not Mdm2.

Sarcoma	*a+/+p−/−*		*a+/−p−/−*		*a−/−p−/−*		*a−/−p+/−*		*a+/−p+/−*	
subtype	*m+/+*	*m+/−*	*m+/+*	*m+/−*	*m+/+*	*m+/−*	*m+/+*	*m+/−*	*m+/+*	*m+/−*
Hemangio	70%	80%	50%	56%	41%	44%	6%	12%	13%	27%
Soft Tissue	20%	18%	40%	33%	35%	44%	67%	65%	38%	18%
Histiocytic	10%	9%	10%	11%	12%	0%	22%	12%	25%	18%
Osteo	0%	0%	0%	0%	12%	11%	0%	6%	25%	36%
Carcino	0%	0%	0%	0%	0%	0%	6%	6%	0%	0%

Percentage of total sarcomas from the specified genotype with the specific sarcoma subtype.

Comparisons between *Mdm2+/−* and *Mdm2+/+* mice revealed differences in tumor spectrum in some, but not all, of the different *Arf/p53* genotypes (Table 2). There was a similar tumor spectrum in *Mdm2+/−Arf*+/+*p53−/−* and *Mdm2+/+Arf*+/+*p53−/−* mice and between *Mdm2+/−Arf*−/−*p53+/−* and *Mdm2+/+Arf*−/−*p53+/−* mice. However, a greater percentage of *Mdm2+/−Arf*+/−*p53−/−* mice developed lymphomas than *Mdm2+/+Arf*+/−*p53−/−* mice, but they had a similar rate of sarcoma development. More lymphomas than sarcomas emerged in *Mdm2+/−Arf*−/−*p53−/−* mice, whereas the opposite occurred in *Mdm2+/+Arf*−/−*p53−/−* mice. The differences in the number of sarcomas (8 in 19 mice versus 15 in 20 mice; p = 0.037, chi-squared test) and lymphomas (14 in 19 mice versus 8 in 20 mice; p = 0.034, chi-squared test) that developed in *Mdm2+/−Arf*−/−*p53−/−* mice compared to *Mdm2+/+Arf*−/−*p53−/−* mice were statistically significant. Notably, in *Mdm2+/−Arf*+/−*p53+/−* mice, there was a decreased percentage of sarcomas and an increased percentage of carcinomas as compared to *Mdm2+/+Arf*+/−*p53+/−* mice. Specifically, 20% of the malignancies that emerged in *Mdm2+/−Arf*+/−*p53+/−* mice were carcinomas, whereas only 6% were carcinomas in *Mdm2+/+Arf*+/−*p53+/−* mice. Interestingly, billiary and pancreatic carcinomas arose in *Mdm2+/−Arf*+/−*p53+/−* mice and not in the *Mdm2+/+Arf*+/−*p53+/−* mice. The difference in the number of sarcomas that developed in the *Mdm2+/−Arf*+/−*p53+/−* mice (9 of 15 mice) compared to the *Mdm2+/+Arf*+/−*p53+/−* mice (15 of 16 mice) was statistically significant (p = 0.0247, chi-squared test). Although there were strong trends towards different tumor spectrums between *Mdm2+/−* and *Mdm2+/+* mice of various *Arf/p53* genotypes with some approaching statistical significance, only the three comparisons described above and as presented in Table 2 reached statistical significance. Therefore, Mdm2 haploinsufficiency significantly alters tumor spectrum toward lymphoma and away from sarcoma in specific *Arf/p53* genotypes, but only modestly influences or has no effect on tumor spectrum in other *Arf/p53* genotypes.

All genotypes developed more than one tumor type at a similar frequency, with one exception (Table 2). Similar to a previously published report [14], the *Mdm2+/+Arf*+/+*p53−/−* mice primarily developed one tumor type with only 2 mice (11%) having two primary tumors, whereas the *Mdm2+/−Arf*+/+*p53−/−* mice and all other genotypes developed two tumor types with a frequency of 18–29%. *Mdm2+/−* and *Mdm2+/+* mice for each *Arf/p53* genotype had analogous rates of multi-primary tumor development and a similar tumor spectrum. Mice that developed a lymphoma and also a sarcoma were the most common multi-primary malignancies (Table 2). Rarely did mice develop three primary tumors, and no mouse developed four primary cancers. The data indicate an Mdm2 haploinsufficiency did not alter the number of primary tumors that developed in mice irrespective of *Arf/p53* genotype.

Notably, specific genotypes of mice developed one type of sarcoma over another more frequently. For example, sarcomas in *Arf*+/+*p53−/−* mice, regardless of *Mdm2* genotype, were predominantly hemangiosarcomas (Table 3). With loss of one or both alleles of *Arf*, *p53−/−* mice developed more soft tissue sarcomas and less hemangiosarcomas. Interestingly, *Arf*−/−*p53+/−* mice preferentially developed soft tissue sarcomas over all other sarcomas and were the only genotype where carcinosarcomas emerged (Table 3). These data suggest loss of Arf allows for soft tissue sarcomas to emerge more readily, whereas with loss of p53, hemangiosarcomas dominated. *Mdm2+/+Arf*+/−*p53+/−* mice developed a spectrum of sarcomas with a distribution between hemangio, histiocytic, osteo, and soft tissue sarcomas (Table 3). *Mdm2* heterozygosity did not alter the type of sarcomas that developed compared to those that arose in the *Mdm2+/+* matched genotypes except for the *Arf*+/−*p53+/−* mice. There was a trend, although not statistically significant, that *Mdm2+/−Arf*+/−*p53+/−* mice developed more hemangiosarcomas and less soft tissue sarcomas, whereas *Mdm2+/+Arf*+/−*p53+/−* mice developed more soft tissue sarcomas and less hemangiosarcomas (Table 3). Notably, these data indicate Mdm2 levels did not broadly contribute to the type of sarcoma that emerged with the exception of *Arf*+/−*p53+/−* mice, but instead, Arf and p53 levels primarily dictated the sarcoma tumor subtype that developed.

## Discussion

An intact Arf-Mdm2-p53 pathway is critical to prevent tumorigenesis. However, expression levels of these genes can vary normally due to environmental and genetic factors. Moreover, levels of Arf, Mdm2, and/or p53 are typically altered in cancer cells, which contributes to the development and/or progression of the malignancy. In recent years, it has become appreciated that even small changes in the levels of Mdm2 and/or p53 may alter an individual’s susceptibility to tumor development and possibly the type of tumors that emerge. For example, a single nucleotide polymorphism (SNP) in the promoter of Mdm2 (SNP309), leading to increased transcription, or codon 72 of p53, altering protein stability or function, can in certain circumstances increase a person’s susceptibility to tumor development [17], [18]. Our genetic mouse studies reveal the significance of *Arf*, *Mdm2*, and *p53* gene dosage on the rate of tumor development and the types of tumors that arise, and importantly, highlight the critical role a small decrease in Mdm2 has on these processes. Specifically, data here, which are consistent with our previous study [10], show that lower levels of Mdm2 due to *Mdm2* heterozygosity were protective against tumors initiated from an Arf deficiency. Previously, it was shown that reduced levels of Mdm2 in *Mdm2* hypomorphic mice inhibited colon tumor development from loss of adenomatous polyposis coli (*Apc*) [19]. Importantly, our data here also show reduced Mdm2 levels inhibited tumor development in the context of *p53* heterozygosity regardless of *Arf* status. However, loss of *p53* was dominant, and dictated the rate of tumor development independent of *Mdm2* haploinsufficiency. *Mdm2* heterozygosity also significantly altered tumor spectrum in some of the genotypes of mice. Therefore, our data demonstrate that altering Mdm2 levels can have a profound impact on tumorigenesis, and that this was dependent on p53 and independent of Arf. As personalized medicine comes of age, levels of Arf, Mdm2, and p53 and how they interact and function with respect to each other under various conditions in different tissues will be vitally important in surveillance and treatment decisions for a variety of diseases. By utilizing a mouse genetics approach, the data obtained increases understanding of the interplay between Arf, Mdm2, and p53 in tumorigenesis and provides novel insights into the critical role of Mdm2.

Although there is agreement that Arf regulates Mdm2 in the presence of hyperproliferative signals from oncogenes, such as Myc, it remains unresolved under what other conditions Arf regulates Mdm2 and what, if any, influence Arf has on Mdm2 in the absence of hyperproliferative signals from oncogenes. Our data here clarify the role of Arf regulation of Mdm2 in tumorigenesis. Oncogenes activate the Arf-Mdm2-p53 pathway, and therefore, cells that have an overexpressed oncogene are exquisitely sensitive to the levels of Arf, Mdm2, and p53. Similar to Myc oncogene-induced tumorigenesis [20], we showed *Mdm2* heterozygosity inhibited tumor development in *Arf*-null mice and in *Arf−/−p53+/−* mice. However, in contrast to Myc-driven tumor development [15], loss of one allele of *Arf* did not rescue the delay in tumor development caused by an Mdm2 haploinsufficiency. This is an important distinction in tumor development, and our results strengthen the argument for an Arf-independent function for Mdm2 in tumorigenesis in the absence of oncogene overexpression. Moreover, it was previously reported that Arf did not regulate Mdm2, and consequently p53, in proliferating tissues during development and under homeostatic conditions [21]. Our results that loss of one or two alleles of *Arf* did not rescue the delay in tumor development caused by Mdm2 haploinsufficiency support and extend these previous findings to tumor development.

Evidence has emerged showing p53 independent functions of Mdm2 in tumor development [22]. However, determining the extent of p53 involvement in Mdm2 function has, and continues to be, very difficult, due to the overriding phenotypes that emerge in the absence of p53. For example, Mdm2 overexpression in the context of *p53* deletion does not alter tumor latency but does change tumor spectrum [13]. In a previous study, loss of one but not both alleles of *Mdm2* led to an increase in the number of primary tumors, sarcoma development, and a slight (6 day) statistically significant delay in tumorigenesis in *p53*-null mice [14], suggesting a p53-independent consequence of Mdm2 haploinsufficiency. Similarly, we observed an analogous increase in the number of primary tumors in *Mdm2+/−p53−/−* mice. In contrast, we did not detect effects of Mdm2 haploinsufficiency on tumor latency or a bias towards sarcomas in the absence of p53. This may be due to the background differences between the mice of the two studies, since the mice in this current study already had twice the incidence of sarcomas compared to the previous study [14]. Our results on the *Mdm2+/−p53−/−* mice were consistent with another study that showed no change in tumor latency and minor alterations in tumor spectrum compared to *p53−/−* mice [23]. We did detect a significant increase in lymphomas and a decrease in sarcomas due to an Mdm2 haploinsufficiency in two of the genotypes (*Mdm2+/−Arf−/−p53−/−* and *Mdm2+/−Arf+/−p53−/−*) studied. We also observed an increase in carcinomas in *Mdm2+/−Arf+/−p53+/−* mice. In addition, we detected a modest effect on the type of sarcoma that developed in *Mdm2+/−Arf+/−p53+/−* mice. Therefore, alterations in Mdm2 levels can predispose mice to developing certain types of cancers, and this can be independent from p53.

Mdm2 and Arf have been shown to cooperate during tumorigenesis. Specifically, the Donehower group reported that overexpression of Mdm2 and loss of *Arf* cooperated to accelerate tumorigenesis [11], suggesting that suppression of p53 and inactivation of Arf have independent effects in tumor development. Consistent with these results, our data show that loss of both *Arf* and *p53* accelerated the rate of tumorigenesis over that of *p53*-null only mice. Our results also support data that Arf and p53 can function independent of each other in tumor development [12], [16]. However, our data are in contrast to *Weber et al.* that biallelic deletion of both *p53* and *Arf* have tumor latencies analogous to deletion of both alleles of *p53* alone [12]. It is currently unclear why our results differ from those of *Weber et al.*, but genetic background is a likely explanation. The mice used in the *Weber et al.* study were a mixed C57Bl/6/129Sv colony that was not inbred. Our colony, which originated with mice from Dr. Sherr, is an inbred colony (7+ years of inbreeding) that has a stable 64% C57Bl/6 and 36% 129Sv mixed background that is inherited. It appears that in this background, the acceleration in tumor development is revealed. Our data may also indicate that there are tumor modifying genes that cooperate with *Arf* loss on this inbred mixed strain that may explain the difference from the previous study. Overall, our studies reveal new insights into the Arf-Mdm2-p53 pathway in tumorigenesis, and future studies will continue to unravel the complicated interplay of Arf, Mdm2, and p53 in tumor development.

## Materials and Methods

### Mice

*p53+/−Mdm2+/−* (C57BL/6×129Sv) mice were originally obtained from Dr. Guillermina Lozano (MD Anderson Cancer Center), and *Arf*-null mice (C57BL/6×129Sv) were originally provided by Drs. Martine Roussel and Charles J. Sherr (St. Jude Children’s Research Hospital). *Arf−/−* mice were crossed to *p53/Mdm2* heterozygous mice and inbred over 7 years to generate Arf/Mdm2/p53 deficient mice with a stable mixed C57BL/6/129Sv background. All experimental mice described in this manuscript were generated from intercrossing this inbred strain of mice. Microsatellite analysis showed that the mice were a stable 64% C57Bl/6 and 36% 129Sv. All mice in the study were carefully monitored and were humanely sacrificed when signs of illness or tumors were detected. At necropsy, tissues were collected and formalin fixed for histological analysis (see below).

### Histopathology

Organs and tissues from mice were collected, fixed in 10% buffered formalin, and embedded in paraffin. A blinded sampling of mice from each genotype with attention to equal female to male ratios for each *Mdm2+/+* and *Mdm2+/−* set was chosen for pathological/histological analysis. Embedded tissues from the chosen mice were sectioned and stained with Hematoxylin and Eosin (H&E). Dr. Kelli Boyd, a Board Certified Veterinary Pathologist, evaluated the H&E stained sections of tissues and made the diagnosis for each mouse.

### Statistical Analysis

Kaplan-Meier analysis was performed for all mouse crosses and log-rank tests were used to determine statistical significance in the difference in survival between matched cohorts. A 2×2 contingency table Chi-squared test was performed to determine significant differences in the tumor types that arose in each of the *Mdm2+/+* and *Mdm2+/−* genotypes.

### Ethics Statement

Research involving mice followed all institutional, state, and federal rules and regulations and was approved by the Vanderbilt University Institutional Animal Care and Use Committee (protocols #M/06/271 and #M/09/233).
